# Mini Fragment Locking Compression Plate Fixation of a Rare Case of Displaced Medial End Clavicle Fracture

**DOI:** 10.7759/cureus.57743

**Published:** 2024-04-06

**Authors:** Supriya Pokle, Shivappa Devarmani, Swaroop Solunke, Pratik T Gundecha

**Affiliations:** 1 Ophthalmology, Dr. D. Y. Patil Medical College, Hospital and Research Centre, Pune, IND; 2 Orthopaedics, Dr. D. Y. Patil Medical College, Hospital and Research Centre, Pune, IND

**Keywords:** open reduction and internal fixation, displaced fracture, vascular injury, locking compression plating, medial clavicle fracture

## Abstract

Clavicle fractures at the medial end are very rare. Even in cases where there is severe displacement, such fractures have usually been managed nonoperatively. Yet, there are many patients who remain symptomatic over a year following injury, and the non-union rate is also high. Operative intervention for displaced clavicle fractures of the medial end has been more common in the past decade. The possibility of iatrogenic injury due to the near proximity of critical vascular structures continues to be a concern. This case report describes the management of a rare displaced medial end clavicle fracture in a young male. The patient is a 28-year-old male who came with a week-old displaced medial end left clavicle fracture. On examination, tenting of skin was seen over the medial end clavicle region. CT angiography of the left upper limb was performed to check the vascular structures in relation to the fracture, as there remain concerns about the close proximity of underlying vascular structures and the potential for iatrogenic damage. A vascular surgeon was kept on standby during the surgery. The patient was taken up for surgery after a pre-anesthetic checkup and open reduction and internal fixation was done with a 2.4-mm system mini fragment locking compression plate over the anterior surface of the clavicle. The surgery was uneventful, and the patient had a good clinical and radiological outcome postoperatively.

## Introduction

Clavicle fractures at the medial end are uncommon, making for about 2-6% of all clavicle fractures [1]. They have been linked to multi-system injuries and high-energy trauma [2]. Middle-aged men are primarily seen with these fractures after road traffic accidents [2]. Even in cases where there is severe displacement, such fractures have usually been managed nonoperatively [3,4]. Yet, there are reports that up to 50% of patients remain symptomatic over a year following injury, and the non-union rate is close to 15%, indicating that nonoperative management of displaced clavicle fractures of the medial end is often ineffective [4-6]. Operative intervention for displaced clavicle fractures of the medial end has been more common in the past decade. According to the literature, there are no standard surgical procedures for the treatment of the displaced medial clavicle fracture. Many surgical approaches have been described, including Kirschner wire, screw, and T-plate fixation. However, the fixation failure rate was high when using the Kirschner wire and screw [7]. With the T-plate method, the problems of fragment distraction and screw pull-out were common [8]. Recently, several studies have evaluated the treatment procedure for medial clavicle fracture with a locking plate [9]. The possibility of iatrogenic injury due to the near proximity of critical vascular structures continues to be a concern [10,11]. While there have been outstanding results reported with a variety of plate fixation techniques, implant discomfort and fixation failure are common [9,12].

## Case presentation

**Figure 1:**
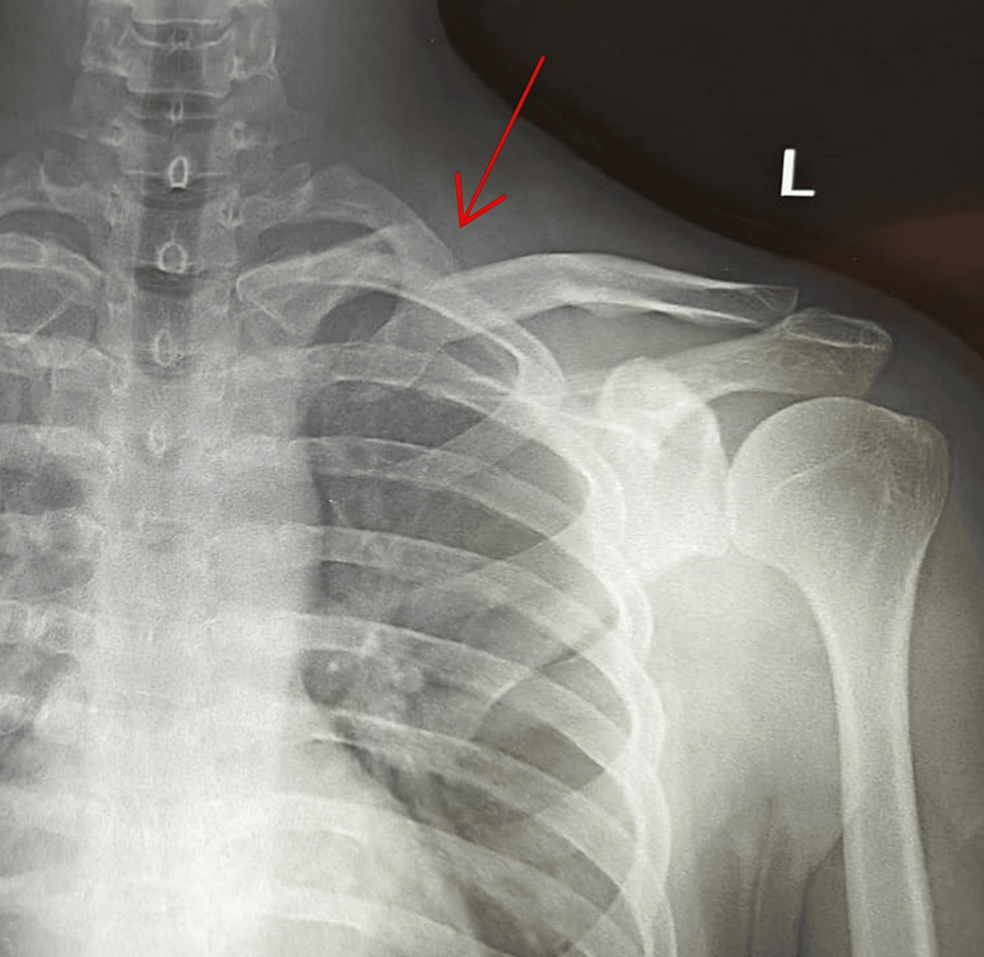
Left shoulder AP view showing displaced medial end clavicle fracture (Edinburgh classification type 1 B1: displaced extra-articular) Red arrow indicating the fracture site AP: anterior-posterior

**Figure 2:**
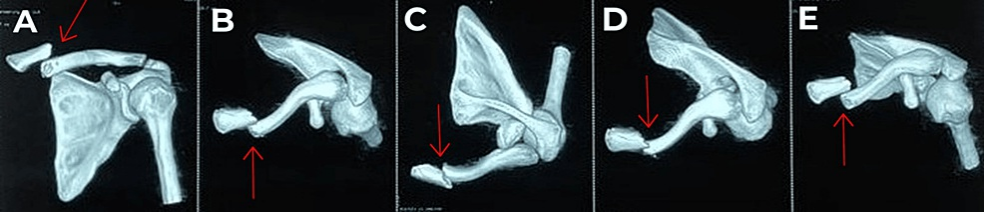
3D CT showing extra-articular displaced medial end clavicle fracture Figures A, B, C, D, and E show multiplanar 3D views of the fracture Red arrows indicating the fracture site 3D CT: three-dimensional computed tomography

A 28-year-old male who is right-handed and has no known medical conditions or surgical history presented to our facility complaining of pain and swelling over his left shoulder for a week. He had a history of road traffic accident (RTA) a week back. Clinical examination showed skin tenting and tenderness over the medial end of the clavicle with no open wound. There was no distal neurovascular deficit. A left shoulder anterior-posterior (AP) view X-ray (Figure 1) showed left medial end clavicle fracture (Edinburgh classification type 1 B1: displaced extra-articular) with the medial fragment displaced superiorly. Left shoulder 3D CT was also done to rule out any intra-articular extension of the fracture (Figure 2).

The diagnosis was missed at the outside facility where the patient was taken immediately after the RTA. However, persistent pain brought the patient to our facility for further treatment. An arm sling was provided for support, and the patient was admitted to the hospital as he was planned for operative management. All necessary pre-op investigations were carried out.

**Figure 3:**
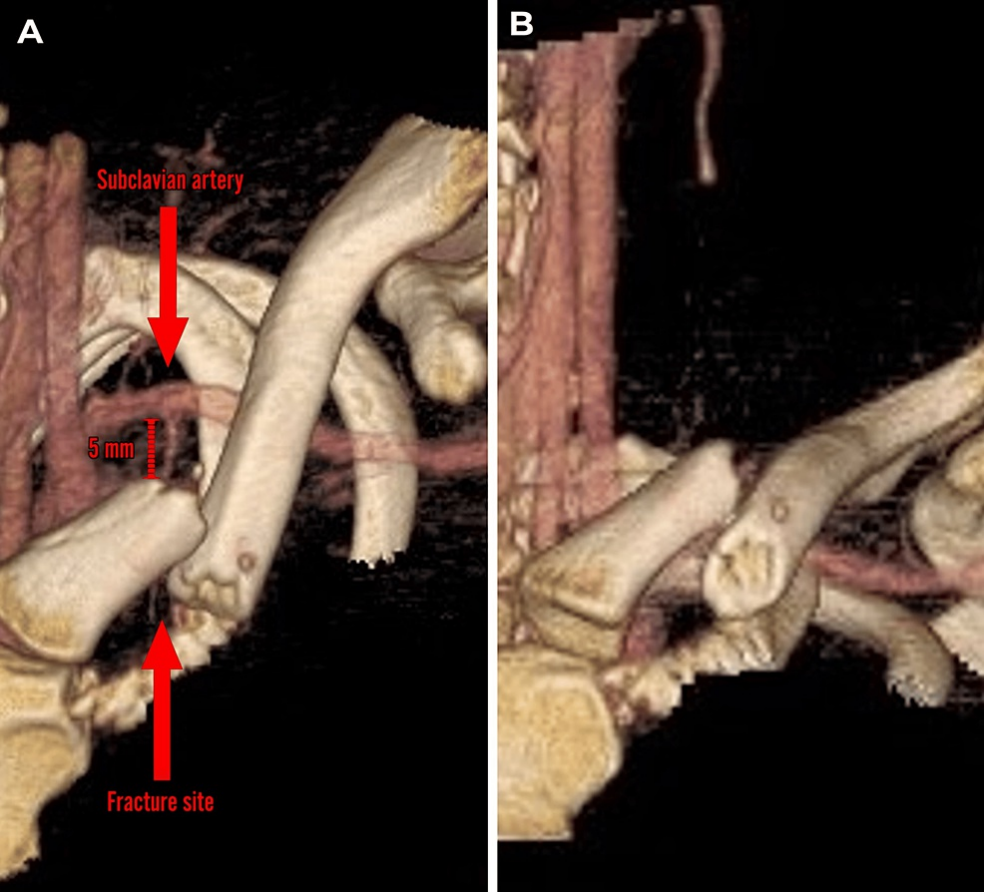
CT angiography of the left upper limb showing the fracture in close proximity to the subclavian artery (approximately 5 mm posterior to the medial fragment) Figure A shows the fracture in cephalo-caudal view where the medial fragment is displaced posteriorly. Figure B shows the fracture in anterior-posterior view where the medial fragment is displaced superiorly

As there are chances of injury to the vascular structures like subclavian vessels in medial end clavicle fractures, a left upper limb CT angiography was done to check the vascular structures in relation to the fracture. It showed the subclavian artery at a distance of approximately 5 mm posterior to the medial fragment which is displaced superiorly and posteriorly (Figure 3A, 3B).

Pre-anesthetic checkup was done, and fitness was given for surgery under general anesthesia for open reduction and internal fixation of the fracture with plating. We explained to the patient and his family the complications such as malunion, non-union, and neurovascular injury.

**Figure 4:**
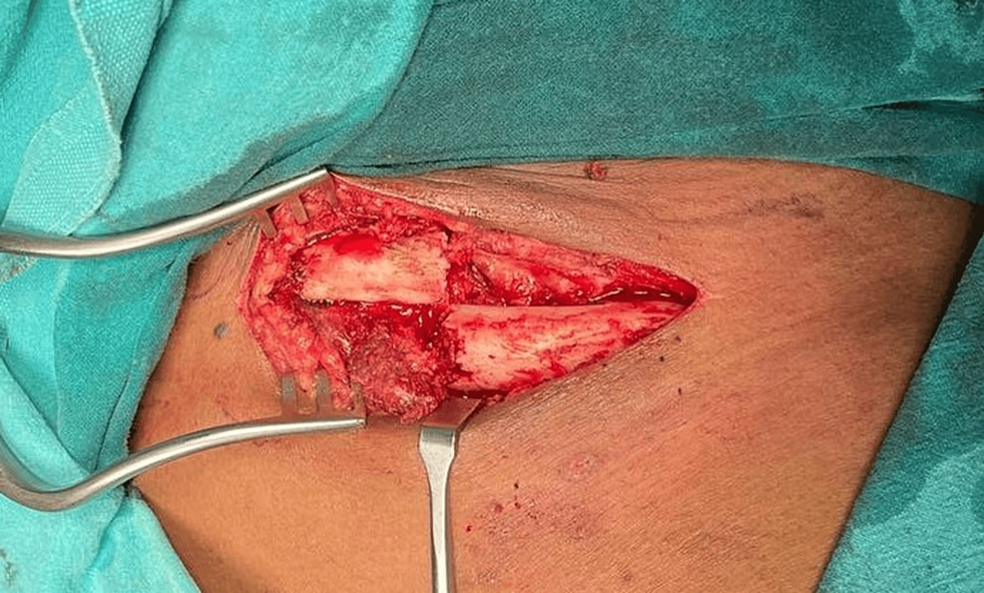
Intraoperative picture showing the fracture site via an anterior approach

**Figure 5:**
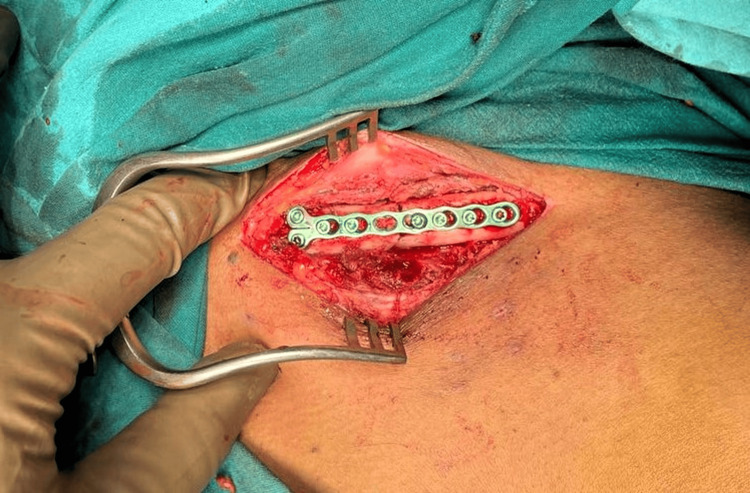
Intraoperative picture showing fracture reduction and fixation with seven-holed mini fragment 2.4-mm system LCP over the anterior surface of the clavicle LCP: locking compression plate

**Figure 6:**
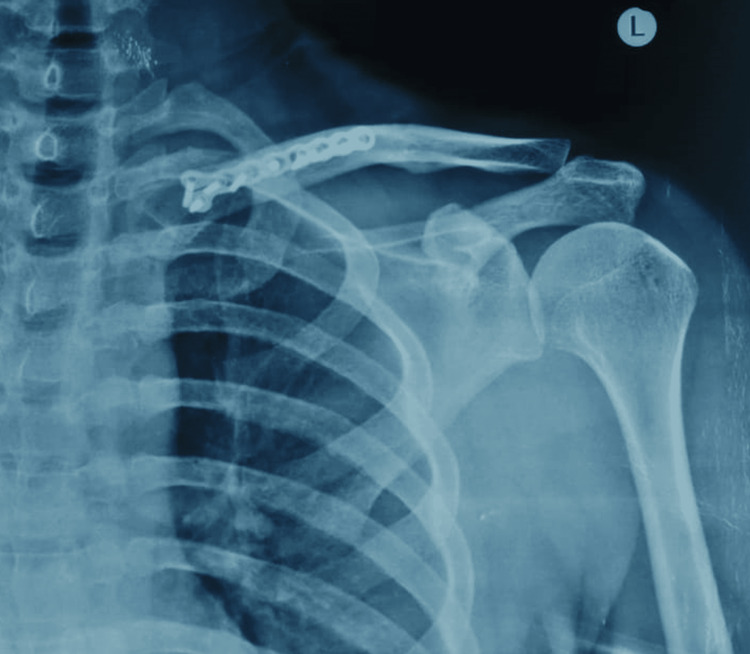
Postoperative anterior-posterior view of the left shoulder showing reduction of the fracture

The patient was preoperatively evaluated for general anesthesia on the day of the procedure. Preoperative antibiotics were administered. The patient was taken in a beach chair position. Scrubbing, painting, and draping were done. The anterior approach was taken, with a 6-cm oblique incision just at the inferior border of the clavicle. The platysma was divided and the dissection taken laterally making sure the sternocleidomastoid wasn't affected. Then the fracture site was exposed. The fracture was reduced using bone-holding forceps, and the plate was fixed anteriorly with four screw purchase on either side of the fracture (four locking cortical screws on the medial side and one non-locking cortical and three locking cortical screws on the lateral side) along with an inter-fragmentary cortical screw taking care not to injure any underlying vessels (Figure 4). A seven-holed mini fragment 2.4-mm system locking compression plate was used (Figure 5). The reduction was satisfactory. Closure was carried out in a standard way, and an arm sling was given for support. There was no neurovascular deficit post-surgery. Postoperative left shoulder AP view X-ray showed the reduction of the fracture (Figure 6).

**Figure 7:**
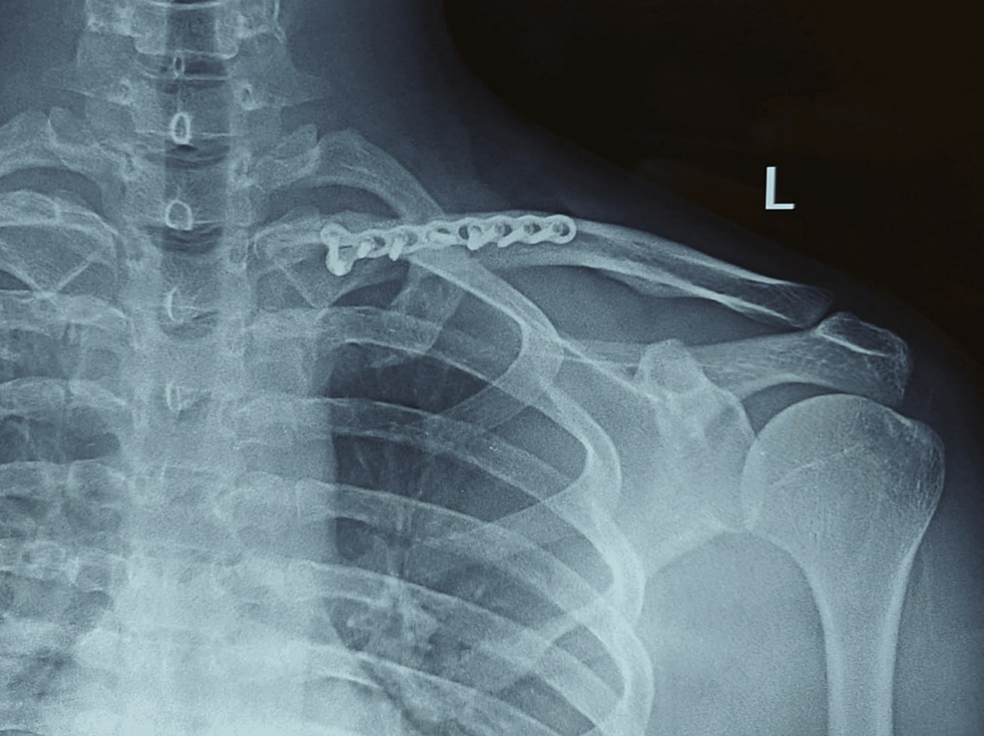
Five months post-surgery follow-up, anterior-posterior view of the left shoulder showing signs of fracture union

**Figure 8:**
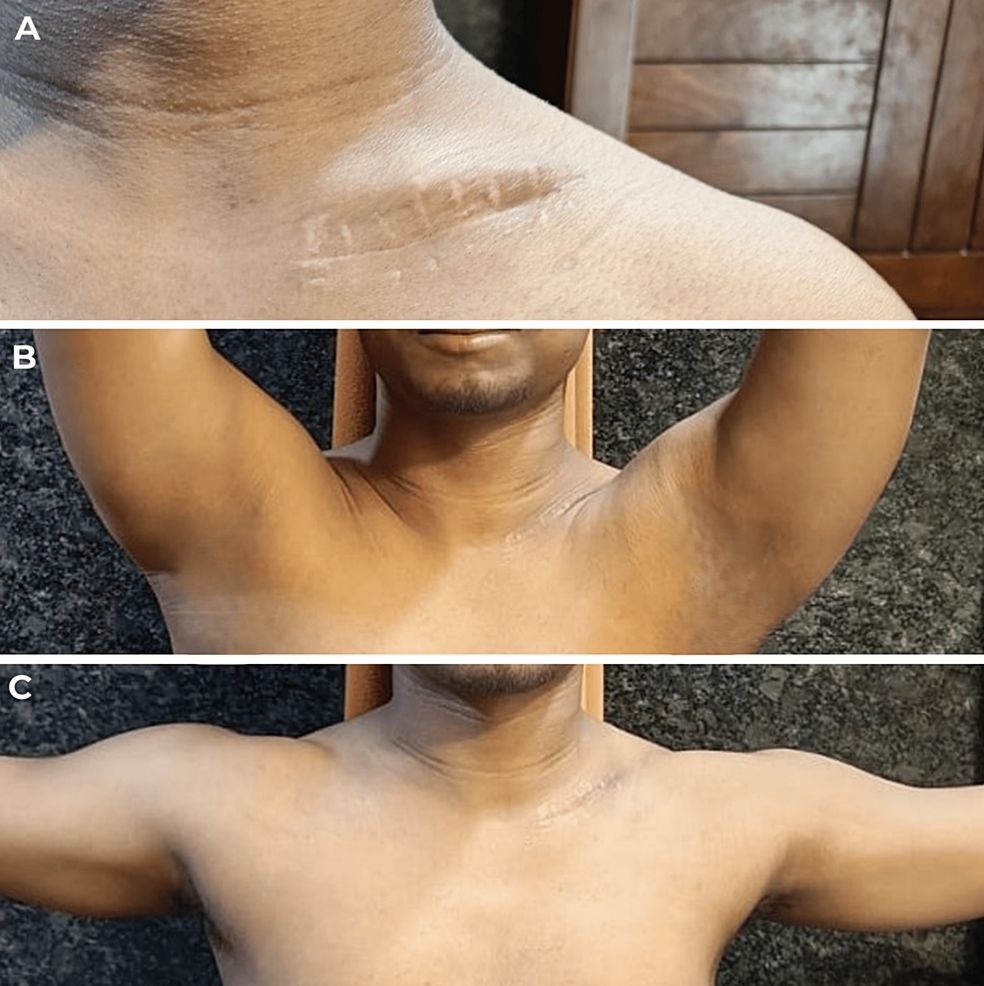
Clinical pictures at five months follow-up showing satisfactory range of motion of the shoulder Figure A shows healed surgical scar by primary intention. Figure B shows satisfactory forward flexion of the shoulder. Figure C shows satisfactory abduction of the shoulder

Following surgery, the patient was examined at two and four weeks. Following a two-week checkup, the suture was removed. At five months follow-up, an X-ray was done to check the fracture union. The X-ray (Figure 7) showed good signs of fracture union, and clinical examination showed satisfactory shoulder range of motion (Figure 8) without any implant irritation. The patient was able to perform all daily activities.

## Discussion

Medial end clavicle fractures have traditionally been considered to be rare. A number of studies have found the instance of about 2-6% [1]. However, these have been retrospective studies looking at X-rays. The problem with X-rays is it's not always easy to see medial end clavicle fractures. Similarly, in our case, the fracture was missed at the first instance in the X-ray taken on the day of trauma at another facility. A CT scan gives better chances of diagnosing medial end clavicle fractures. A more recent study by Throckmorton and Kuhn which looked at CT scans found that the actual instance of medial end clavicle fractures was nearly 10%, with mid-shaft fractures being the most at 63.3% and lateral end accounting for about 20% [4]. The management of medial end clavicle fractures has usually been nonoperative [3,4]. Yet, there are reports that up to 50% of patients remain symptomatic over a year following injury, and the non-union rate is close to 15%, indicating that the nonoperative management of displaced clavicle fractures of the medial end is often ineffective [4-6].

We chose to operate on this fracture as the patient was a young male with complaints of skin tenting due to the displaced fracture which caused him cosmetic problems. The patient was explained about the conservative and operative management along with the pros and cons before proceeding with the surgery.

When considering plate fixation of the medial end clavicle, it's important to remember the anatomy. Lying behind the medial end of the clavicle are a number of great vessels, particularly the subclavian vein and artery. The most appropriate way to treat a long bone fracture is using a plate, and there are a number of plates that are produced according to clavicle anatomy and also pre-contoured plates [13]. However, very few screws can be fixed on the medial side of the medial end clavicle fracture. This would not be sufficient to fix the fracture. So traditional straight plates aren't going to give us sufficient medial hold for the fracture. The next option might be to consider a lateral clavicle plate which has got a flared end with multiple screws to try and fix a fracture. It fits anatomically on the lateral end of the clavicle [14]. However, when we bring the plate to the medial end, it needs to be contoured through 90 degrees to fix it [15]. But this plate was bulky for the patient due to the small dimensions of the clavicle as the patient was short-statured. As the patient was a young male, it would cause a cosmetic problem as well as chances of wound dehiscence and hardware irritation. There is one further issue with putting a plate on the superior surface of the medial end of the clavicle. It is the insertion of the clavicular part of the sternocleidomastoid. This inserts for the first 2 cm at the superior surface of the medial end of the clavicle. When we put a plate onto the superior aspect of the clavicle, we're going to have to take off the whole of the insertion of the sternocleidomastoid. So, trying to fix medial end clavicle fractures with a plate and screws on the superior surface is going to be difficult. The other option is to consider the anterior surface upon which the plate fits nicely, and we can put our screws in anterior to posterior. However, the concern is that the subclavian vessels lie directly behind. There also we only really get two screws into the medial end of the clavicle with traditional plates which may or may not be sufficient. So, we used a seven-holed mini fragment 2.4-mm system locking compression plate which gives four screw purchases on either side of the fracture along with an inter-fragmentary screw. And this was the longest possible plate in mini fragment system which could be used. This plate served our purpose of preventing wound dehiscence and hardware irritation in the patient who had small clavicular dimensions as the patient was short-statured. And to address the issue of vascular structures lying behind the clavicle, we got CT angiography done to see the vascular structures in relation to the clavicle. Also, a vascular surgeon was kept on standby in case any vascular injury occurs. The mean distance of the subclavian vein is less than 10 mm and the subclavian artery is more than 10 mm with respect to the medial end of the clavicle [16]. Hence, careful drilling was done to prevent any vascular injury, as the subclavian vessels were in close proximity to the displaced fracture, in this case approximately 5 mm posterior to the medial fragment.

We suggest operating medial end clavicle fractures taking into account the age and physical and cosmetic requirements of the patient after explaining the pros and cons of conservative and operative management. The risks of conservative management include non-union, malunion, persistent pain, and skin tenting, whereas vascular injury, wound dehiscence, and implant irritation pose major problems in operative management as described earlier. The mini fragment plate chosen in this case serves our purpose of preventing wound dehiscence and implant irritation, also resulting in excellent radiological and functional recovery.

## Conclusions

We gathered this case to describe this uncommon complicated injury and our management choices, given the rarity of medial clavicle fractures linked to substantial displacement and the paucity of published examples in the literature about their surgical treatment and sequelae. We used the mini fragment system locking plate in this case taking into account the dimensions of the clavicle and the physical and cosmetic requirements of the patient. Thus, it appears that the selected course of treatment will result in an excellent radiographic and functional recovery, also preventing wound dehiscence and implant irritation.
